# Dual Detection of Hemagglutinin Proteins of H5N1 and H1N1 Influenza Viruses Based on FRET Combined With DNase I

**DOI:** 10.3389/fmicb.2022.934475

**Published:** 2022-06-30

**Authors:** Zhiyun Wang, Qiuzi Zhao, Mengqian Huang, Yuqin Duan, Feifei Li, Tao Wang

**Affiliations:** ^1^School of Environmental Science and Engineering, Tianjin University, Tianjin, China; ^2^School of Life Sciences, Tianjin University, Tianjin, China

**Keywords:** hemagglutinin, H1N1, H5N1, FRET, DNase I

## Abstract

Influenza A viruses (IAV) are classified based on their surface proteins hemagglutinin (HA) and neuraminidase (NA). Both pandemic H1N1 (pH1N1) and highly pathogenic avian influenza (HPAI) H5N1 viruses pose a significant threat to public health. Effective methods to simultaneously distinguish H1N1 and H5N1 are thus of great clinical value. In this study, a protocol for detection of HA proteins of both H1N1 and H5N1 was established. Specifically, we designed an aptasensor for HA using fluorescence resonance energy transfer (FRET) strategy combined with DNase I-assisted cyclic enzymatic signal amplification. HA aptamers of H1N1 and H5N1 IAVs labeled with various fluorescent dyes were used as probes. Graphene oxide (GO) acted as a FRET acceptor for quenching the fluorescence signal and protected aptamers from DNase I cleavage. The fluorescence signal was recovered owing to aptamer release from GO with HA protein. DNase I-digested free aptamers and HA proteins were able to further interact with more fluorescent aptamer probes, resulting in increased signal amplification. The limits of detection (LOD) of H5N1 HA and H1N1 HA were 0.73 and 0.43 ng/ml, respectively, which were 19 and 27 times higher than LOD values obtained with the DNase I-free system. The recovery rate of HA protein in human serum samples ranged from 88.23 to 117.86%, supporting the accuracy and stability of this method in a complex detection environment. Our rapid, sensitive, and cost-effective novel approach could be expanded to other subtypes of IAVs other than H1N1 and H5N1.

## Highlights

- A Signal-Amplified Aptamer Sensor Capable of Dual Detecting H5N1 HA and H1N1 HA.
- DNase I Enzyme-Aided Fluorescence Signal Amplification Based on Graphene Oxide-DNA Aptamer.

## Introduction

Influenza A viruses (IAV) belonging to the family Orthomyxoviridae are considered a major threat to public health owing to their wide host range, rapid mutational rates, and capacity to cause epidemics and pandemics worldwide. Hemagglutinin (HA), an antigenic glycoprotein located on the surface of influenza viruses, is responsible for mediating the adsorption of the virus to cell surface receptors (Anderson et al., 2017). IAV are divided into subtypes according to the antigenic differences between neuraminidase (NA) and HA (Schulze et al., 2010). Overall, 18 HA subtypes and 11 NA subtypes have been characterized and many different combinations of HA and NA proteins are possible (Gu et al., 2019). H1N1 influenza virus infection in humans is usually mild (Huo et al., 2019) while H5N1 is a highly pathogenic (HP) strain with a reported mortality rate of about 60% (Yin et al., 2013). Rapid and sensitive simultaneous detection of H5N1 and H1N1 should present an effective solution to manage the influenza pandemic and control the spread of HP viral infections.

Common detection methods for influenza viruses include viral culture, serology, and genetic testing (Gopinath et al., 2014). However, virus cultivation requires trained technicians and complex and expensive equipment (Eisfeld et al., 2014). A serological test is usually an enzyme-linked immunosorbent assay-based test, which is limited owing to expensive reagents and time-consuming processes (Chen et al., 2019), while the polymerase chain reaction test is prone to false-positive results (Miarka et al., 2014). Fluorescence resonance energy transfer (FRET) is a distance-dependent energy transfer process from the excited fluorophore donor to the acceptor, resulting in the quenching of donor molecule fluorescence (Algar et al., 2019). FRET is a widely used spectral tool for the detection of intermolecular interactions (Ardestani et al., 2019). In particular, DNA-based FRET probes have been developed for monitoring homogenous hybridization without solid support and washing procedures, facilitating easy adaptation to automated performance due to rapid and simple detection (Wang et al., 2019; Zhao et al., 2021). Aptamers, generated from the randomized nucleic acid library, are short single-stranded oligonucleotides with specific three-dimensional structures that can form complexes with various target molecules, including small molecules, proteins, and living cells (Wan et al., 2017; Rm et al., 2020). Relative to antibodies, aptamers are easier to prepare and modify, higher in sensitivity, and more suitable for aptasensor development (Jarczewska and Malinowska, 2020).

Graphene oxide (GO) is an oxidized single-atom-thick and two-dimensional carbon material with a large surface area and multivalent structure (Yu et al., 2020). GO strongly binds single-stranded DNA/RNA aptamers through hydrophobic/π-stacking interactions, serving as an efficient quencher for various fluorophores *via* FRET mechanisms (Giust et al., 2018; Niu et al., 2019). Moreover, GO rarely adsorbs folded or double-stranded DNA structures (Yuan et al., 2017). The folded conformation of ssDNA aptamers is restored in the presence of a target, leaving GO and destroying FRET, thus leading to fluorescence recovery (Wang et al., 2018; Su et al., 2019; Sun et al., 2021). In the presence of DNase I, an endonuclease that non-specifically cleaves DNA, the ssDNA aptamer is digested and the target released, which then rebinds more aptamers adsorbed on the GO surface, amplifying the fluorescence signal (Wang et al., 2018).

Prevalent H1N1 contains 2009 pandemic viral genes facilitating human infection (Sun et al., 2020). Fortunately, HP influenza viruses of the H5N1 subtype have not yet acquired the ability to transmit efficiently within the human population. H1N1 and H5N1 have high genetic compatibility and reassortment ability, which greatly enhance the opportunity for viral adaptation in human hosts, raising concerns for the possible generation of pandemic strains (Octaviani et al., 2010). As the symptoms of H1N1 and H5N1 infection are almost identical, our novel dual detection system for the two influenza viruses provides a rapid, sensitive, and economical aptasensor that could be effectively used for systematic surveillance of both pathogenic subtypes to ensure early warning and preparedness for potential future pandemics.

## Materials and Methods

### Apparatus and Chemicals

Hemagglutinin proteins of influenza A H5N1 (H5N1A/Anhui/1/05) and H1N1 (H1N1A/New Caledonia/20/99), the targets for H5N1 and H1N1 AIV detection, were purchased from Sino Biology (Beijing, China). DNA aptamers were synthesized by Shanghai Sangon Biotechnology Co., Ltd. (Shanghai, China) as HA-H5N1 probe: 5′-FAM-TCA TCG ACA CGG GTT CCA GCG ATG TAT AAG AGT GCT ATA GGG TGG CGA TAT GTC CCC-3′ (Shiratori et al., 2014) and HA-H1N1 probe: 5′-ROX-GGC CTA CCG TAG TGT GCG TGG GCA CAT GTT CGC GCC ACC GTG CTA CA AC-3′ (Lao et al., 2014). Aptamer powder was dissolved in TE buffer (pH 7.4, 150 mM NaCl, 10 mM MgCl_2_, 10 mM KCl) to generate a 100 μM stock solution and stored in a dark place at −20°C until use. For experiments, 200 nM working solution was prepared with binding buffer and stored at 4°C.

DNase I, bovine serum albumin (BSA), human serum albumin, and immunoglobin G (IgG) were obtained from Solarbio Life Sciences (Beijing, China). Healthy human serum samples were provided by the Chinese Academy of Inspection and Quarantine. GO was acquired from XFNANO Co., Ltd. (Nanjing, China), and boric acid (H_3_BO_3_), sodium tetraborate decahydrate (Na_2_B_4_O_7_·10H_2_O), sodium chloride (NaCl), and potassium chloride (KCl) from Beijing Chemical Works (Beijing, China). All reagents were of analytical grade or higher, and water used for buffer solutions was prepared using a Milli-Q water system (Millipore Corp., USA) with a resistivity of 18.2 MV cm. Fluorescence spectra were obtained using an EnSpire Multimode Plate Reader (PerkinElmer, Inc., USA).

### Optimization of Fluorescent Receptor Reaction Conditions

#### Optimization of GO Concentration and Quenching Time

H5N1-FAM or H1N1-ROX (200 μl of 200 nM) was mixed with different volumes of 1 mg/ml GO (0, 8, 16, 24, 32, 40, 48, 64, and 80 μl), diluted with BB to a total volume of 800 μl and incubated for 20 min under light avoidance at 30°C. Samples (200 μl/well) were added to a black 96-well plate. Fluorescence intensities of FAM (excitation at 492 nm, emission at 518 nm) and ROX (excitation at 575 nm, emission at 602 nm) were evaluated using a multifunctional microplate reader over a series of reaction time-points (0, 1, 2, 3, 5, 10, 15, and 20 min) at the optimal GO concentration. The optimum quenching time was determined for subsequent experiments.

#### Selection of Blocking Agents and Optimum Concentrations

H5N1-FAM probe (200 nM) and GO (1 mg/ml) were mixed, incubated for 5 min, and treated with PEG800, PEG2000, Tween-80, polymeric 1-pyrene butyric acid (PBA), and MiliQ H_2_O (at a final blocker concentration of 50 nM) to set up five blocking agent groups. After incubation for 10 min, 6.4 μl HA protein of H5N1 (2 μg/ml) was added, and the final volume of the reaction system was set to 800 μl. Solutions were incubated in the dark at 30°C for 30 min. The most effective blocker was determined according to the fluorescence intensity of FAM and the optimum concentration established *via* examination of fluorescence intensities at final concentrations of 0, 10, 20, 50, and 100 nM in the reaction system.

### Cross-Reactivity of Aptamers of HA Proteins From H1N1 and H5N1

Dual probes of H1N1 and H5N1 detection systems targeting HA protein of H1N1 were set up. To this end, fluorescent probes H5N1-FAM and H1N1-ROX (200 μl of 200 nM) were mixed with 40 μl of 1 mg/ml GO and incubated for 5 min, followed by incubation with 16 μl of 1 μM PEG800 for 10 min. Different concentrations of H1N1 HA protein were added to generate final concentrations of 0 (control), 200 ng/ml, 500 ng/ml, and 1 μg/ml, and the fluorescence intensities of FAM and ROX were monitored. Dual probes of H1N1 and H5N1 detection systems targeting HA protein of H5N1 were additionally prepared using the above procedure.

### Feasibility of DNase I in the Detection System

For assessing the amplification capability of DNase I in the GO-aptamer system, five groups, namely, 200 μl of 200 nM H5N1-FAM probe, 300 μl of BB buffer, 40 μl of 1 mg/ml GO, 16 μl of 1 μM PEG800, and 5.3 μl of 5 mg/ml DNase I, were prepared, to which 3.2 μl H5N1 HA protein was added. The fluorescence intensity of the H5N1-FAM probe was measured. The specific reactants of each system are presented in Table 1.

**Table 1:** Composition of the detection systems in different groups.

**Group**	**Compositions of the detection systems**				
	**H5N1-FAM probe**	**GO**	**PEG800**	**DNase I**	**H5N1 HA**
1	+		–	–	–
2	+	+	–	–	–
3	+	+	+	–	–
4	+	+	+	+	–
5	+	+	+	+	+

The optimal DNase I concentration and incubation time were further examined. To establish the optimal dose, fluorescence signals of ROX and FAM at different DNase I concentrations of 0, 5, 10, 15, 20, 25, and 30 U in the detection system were measured. For determination of the optimal incubation time, fluorescence signals at different time points were measured under similar conditions using a fixed DNase I dose of 25 U.

### Dual Assay for HA of H1N1 and H5N1

Dual detection of H1N1 HA and H5N1 HA was performed based on the aptamer-GO-DNase I system. Specifically, fluorescent probes H5N1-FAM (200 μl of 200 nM) and H1N1-ROX were mixed with 40 μl of 1 mg/ml GO and incubated for 5 min at room temperature. The mixture was incubated with 16 μl of 1 μM PEG800 for 10 min as a surface-blocking agent, followed by the addition of 3.3 μl DNase I. A series of concentrations of H5N1 HA and H1N1 HA were added to the GO-aptamer solution and incubated in the dark at 30°C for 45 min. The final concentrations of H5N1 HA used were 0, 0.5, 1, 1.5, 2, 3, 4, 5, 7, 10, 15, and 20 ng/ml and final concentrations of H1N1 HA were 0, 0.5, 1, 1.5, 2, 3, 5, 7, 10, 12, and 15 ng/ml. Other parallel groups were simultaneously set up under the same conditions, except for the absence of DNase I, and fluorescence intensities detected.

### Specificity Test for HA Protein

The experimental conditions for the specificity test for HA were similar to those described above, except different proteins were used. The detection system contained 10 ng/ml of H5N1 HA and H1N1 HA. In the parallel groups, HA protein was replaced with 20 ng/ml NP (nucleoprotein of SFTSV virus), BSA, and IgG, and fluorescence signals were measured.

### Determination of the Influenza Virus Subtype in Serum Samples

Simulated human serum samples were examined as described above. Specifically, healthy human serum was diluted with different subtypes and concentrations of HA and fluorescence intensities are recorded in Table 1.

## Results

### Principle of Dual Detection of H1N1 HA and H5N1 HA

**Figure 1:**
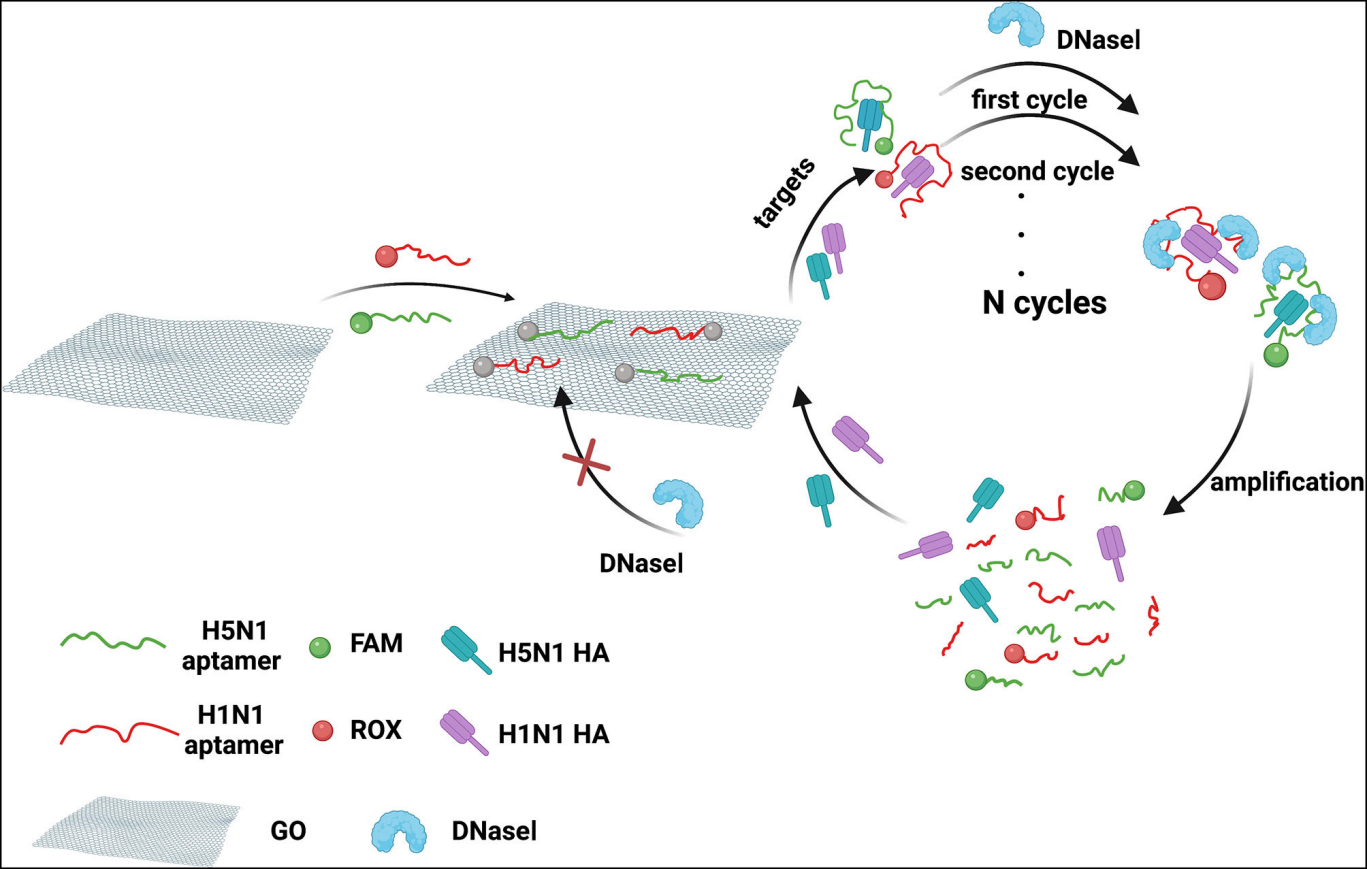
Schematic diagram of dual detection of HA protein from influenza viruses H5N1 and H1N1 based on FRET combined with DNase I.

We developed a homogeneous fluorescence method for dual detection of HA proteins of H1N1 and H5N1 influenza viruses. A schematic representation of the principle is depicted in Figure 1. The red fluorescent dye ROX was used to label the DNA aptamer of HA of the H1N1 influenza virus and the green fluorescent dye FAM was used to target the aptamer of HA of H5N1. GO, a fluorescent acceptor that adsorbs DNA aptamers through a π-π stacking effect was utilized for fluorescence quenching. In the presence of HA, the aptamers formed stable spatial structures and were released from the GO surface, accompanied by fluorescence recovery. To improve detection sensitivity, PEG800 was selected to block the GO surface with the aim of lowering non-specific adsorption. For further amplification of the fluorescence signal, DNase I was introduced into the reaction system, which digested free DNA aptamers outside the GO surface. HA released from aptamers was able to bind fluorescent aptamer probes still absorbed on the GO surface, resulting in further cycles of signal amplification at the same HA concentration. Notably, DNase I could not act on aptamers adsorbed on the surface of GO, i.e., GO protected aptamers from DNase I-induced cleavage. In this system, the concentrations of HA of H5N1 and H1N1 influenza viruses in the sample could be determined by scanning the fluorescence emission signals of FAM and ROX based on the linear relationships between HA concentrations of H1N1 and H5N1 and the corresponding fluorescence intensities of ROX and FAM within a certain range.

### Optimization of GO Concentration and Incubation Time

**Figure 2:**
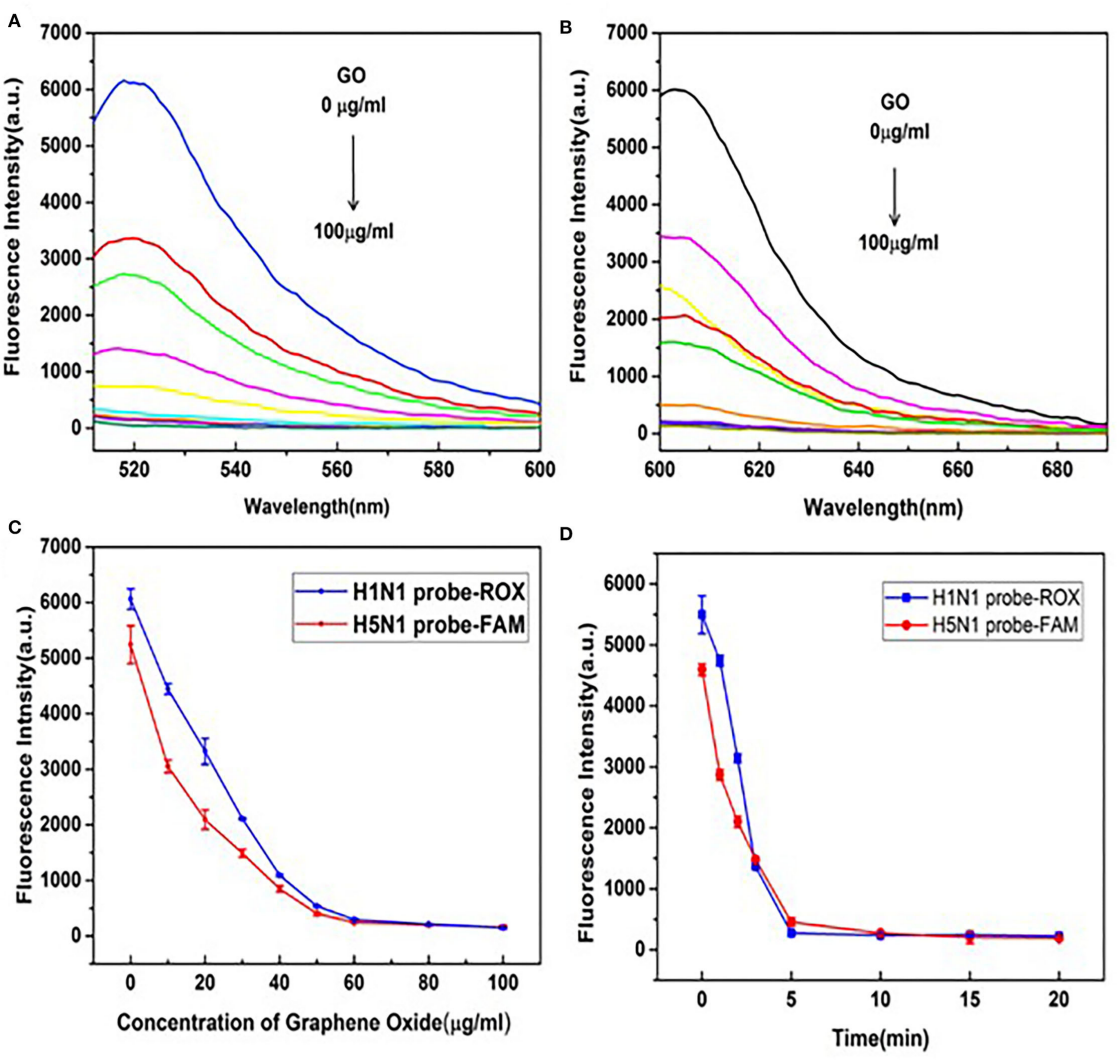
Optimization of GO reaction conditions. **(A)** Fluorescence emission spectral signals of the H5N1-FAM probe. **(B)** H1N1-ROX probe in the presence of different concentrations of GO (0, 10, 20, 30, 40, 50, 60, 80, and 100 μg/ml). **(C)** Changes in trends of fluorescence signals of H5N1-FAM and H1N1-ROX probes in the presence of different concentrations of GO (0, 10, 20, 30, 40, 50, 60, 80, and 100 μg/ml). **(D)** Changes in trends of fluorescence signals at different time points (0, 1, 2, 3, 5, 10, 15, and 20 min) in the presence of GO (50 μg/ml).

To achieve maximum fluorescence quenching and recovery, the GO concentration requires optimization. As shown in Figures 2A,B, with increasing GO concentrations, peak fluorescence spectra of H5N1-FAM and H1N1-ROX showed a downward trend, indicative of quenching. The fluorescence intensities decreased rapidly with increasing concentrations of GO in the range of 10–50 μg/ml, followed by a tendency to level off (Figure 2C). Accordingly, 50 μg/ml GO was selected for subsequent experiments. To establish the optimal incubation time of GO, fluorescence intensities were measured at 0, 1, 2, 3, 5, 10, 15, and 20 min after the addition of GO (Figure 2D). Incubation for 5 min resulted in the highest fluorescence quenching efficiency, after which a plateau was observed. The optimal GO incubation time of 5 min was employed for subsequent experiments.

### Selection and Optimization of Blocking Agents

Single-stranded DNA aptamers labeled with fluorescence dye are rapidly adsorbed by GO, leading to quenching of fluorescence intensity. Ideally, target proteins recognize DNA aptamers, leading to their release due to higher affinity, and ultimately, fluorescence recovery. GO is a heterogeneous two-dimensional carbon nanomaterial containing highly oxidized regions with low DNA adsorption affinity and crystalline carbon regions with high affinity in addition to regions between these two extremes. If DNA aptamers are adsorbed onto high-affinity regions, the release and generation of signals is difficult, and detection sensitivity is significantly reduced. Therefore, blocking the GO surface *via* subtle control of surface forces using specific agents may improve sensor performance. To examine this theory, the utility of blocking agents, such as the small-molecule surfactants Tween-80, polyethylene glycol (PEG2000 and PEG800), and PBA, was further screened.

**Figure 3:**
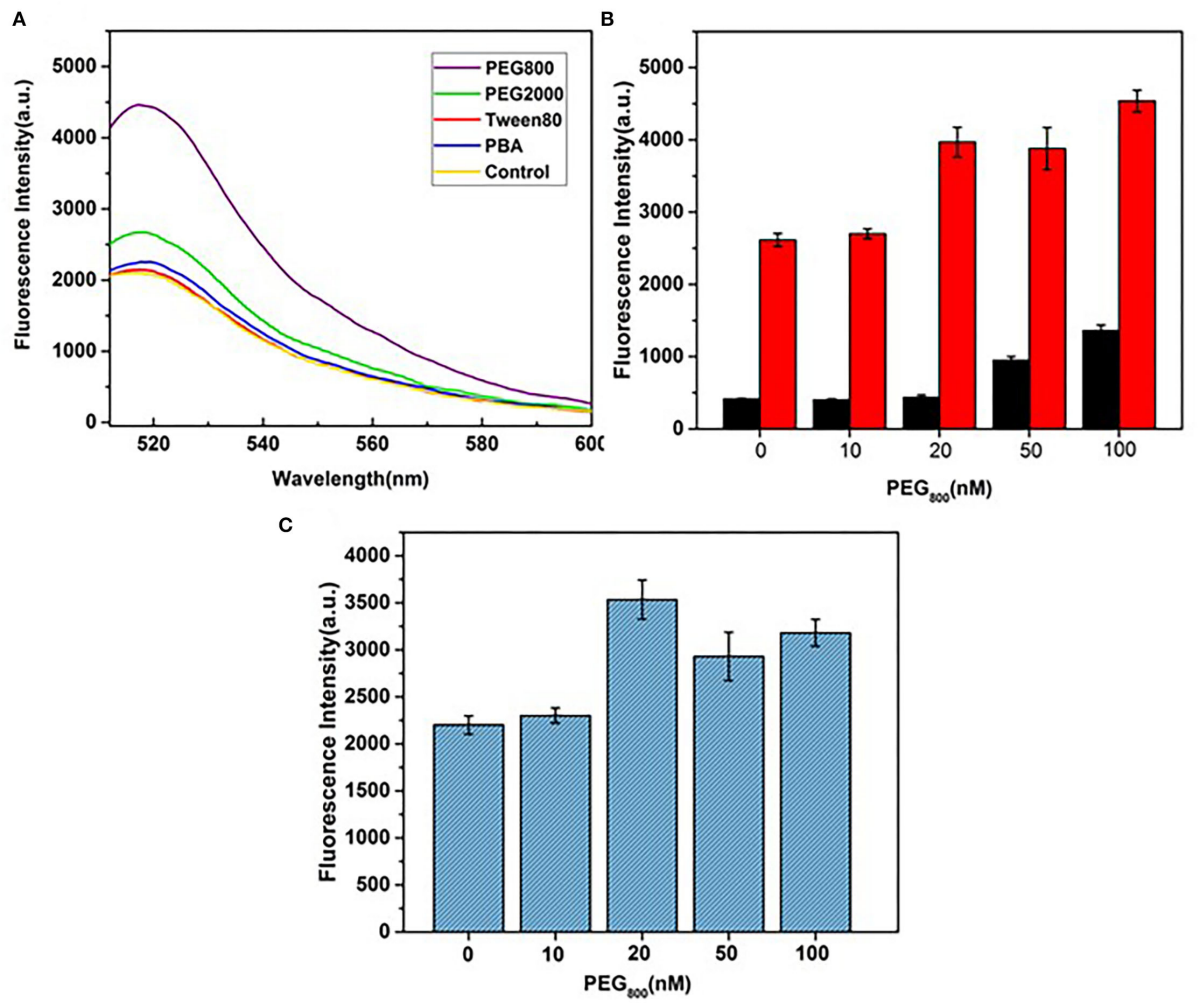
Selection and optimization of blocking agents. **(A)** Fluorescence recovery effects of different types of blocking agents. **(B)** Effects of PEG800 concentration on fluorescence quenching (black bar) without HA and fluorescence recovery (red bar) with HA. **(C)** Effects of PEG800 concentration on fluorescence signals.

As shown in Figure 3B, in the absence of HA, fluorescence intensity remained at a low level with increasing concentrations of PEG800. This blocking agent did not inhibit DNA aptamer adsorption on the GO surface and the fluorescence signal remained quenched as before. In the presence of HA, a more obvious fluorescence recovery was observed with PEG800 and PEG2000 relative to other blocking agents (Figure 3A). The strong hydrophobic interactions of PEG with GO may contribute to lower DNA adsorption capacity. For improvement of detection sensitivity, the concentration of PEG800 that exerts optimal blocking effects is required. To this end, differences in fluorescence quenching and recovery with varying concentrations of PEG800 were compared (Figure 3C). The greatest recovery of fluorescence was achieved at a concentration of 20 nm PEG800, which was thus selected as the optimal concentration for subsequent experiments. Aptamer adsorption on the GO surface was more difficult at higher concentrations of PEG800, leading to reduced sensing performance.

### Cross-Reactivity of HA Probes in Dual Detection of H1N1 and H5N1

**Figure 4:**
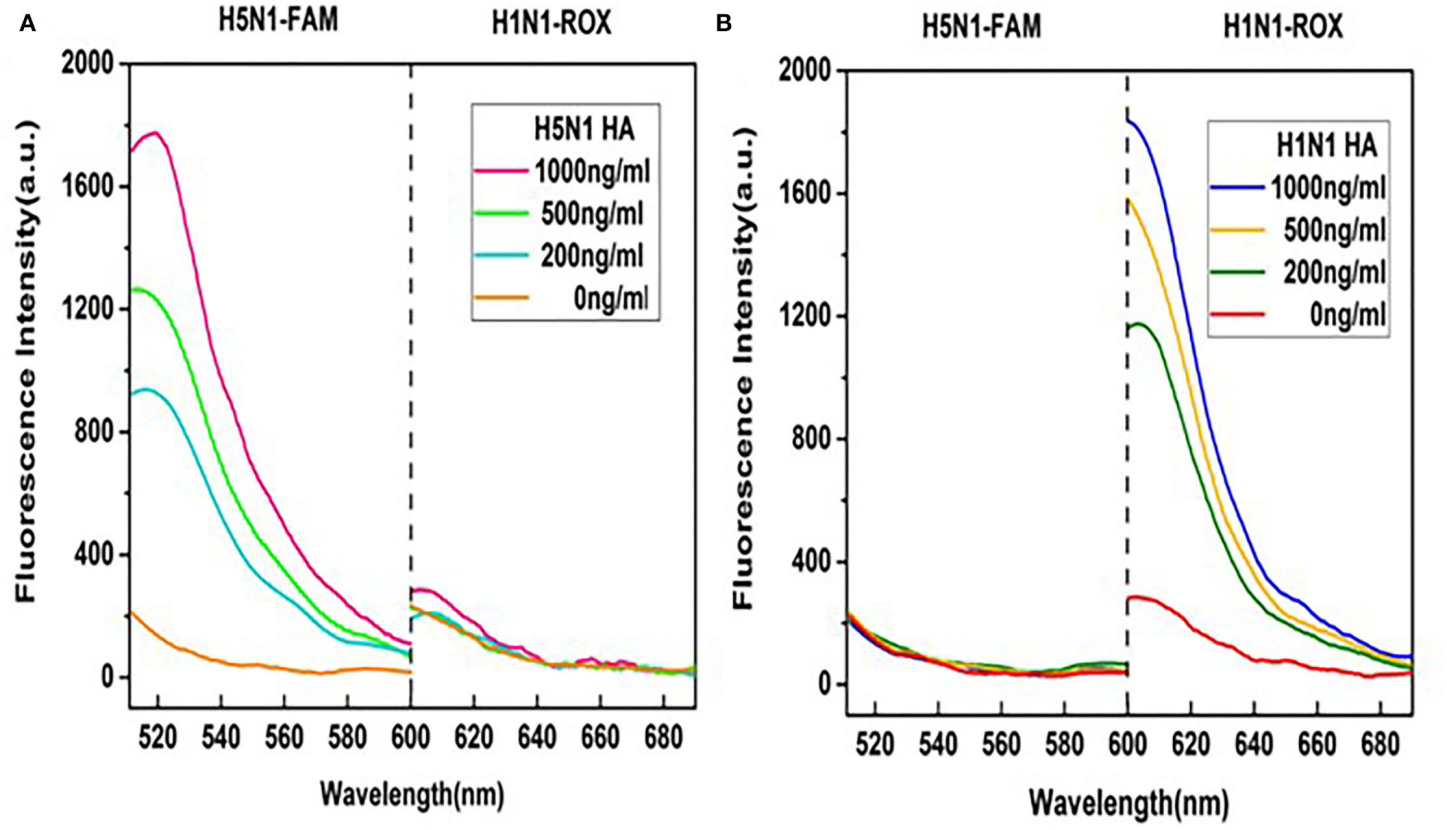
Examination of cross-reactions by monitoring fluorescence spectra of the two probes. **(A)** Different concentrations of HA of H5N1 in the detection system. **(B)** Different concentrations of HA of H1N1 in the detection system. A range of HA protein concentrations (0, 200, 500, and 1,000 ng/ml) was examined at a fixed GO concentration of 50 μg/ml.

The premise of the dual detection reaction system is that no cross-reactions occur between the two pairs of fluorescent probes and their targets. HA probes of H1N1 or H5N1 were thus individually validated in our multiplex detection system. As shown in Figure 4A, in the presence of the aptamer for HA of H5N1, the fluorescence signal of the H1N1-ROX probe was quenched entirely while the fluorescence of the H5N1-FAM probe was recovered. The degree of recovery was positively correlated with the concentration of H5N1 HA. Under similar reaction conditions, in the presence of the aptamer for HA of H1N1, only ROX fluorescence intensity of the H1N1 probe was gradually increased (Figure 4B). Our data indicate that the aptamer sensor shows no cross-reaction between fluorescence probes and HA subtypes.

### Feasibility of GO Quenching and DNase I Cleavage

DNase I is a non-specific endonuclease that can cleave single-stranded DNA, such as aptamers. In the absence of HA protein, the aptamer attached rapidly to the GO surface and was protected from digestion by DNase I. Upon addition of HA, a well-folded HA-aptamer complex was formed and released from the GO surface. The released aptamers were digested by DNase I and free HA recycled to bind further aptamers on GO. Repeated cycles resulted in amplification of the fluorescence signal. Consequently, higher sensitivity performance was achieved using DNase I.

**Figure 5:**
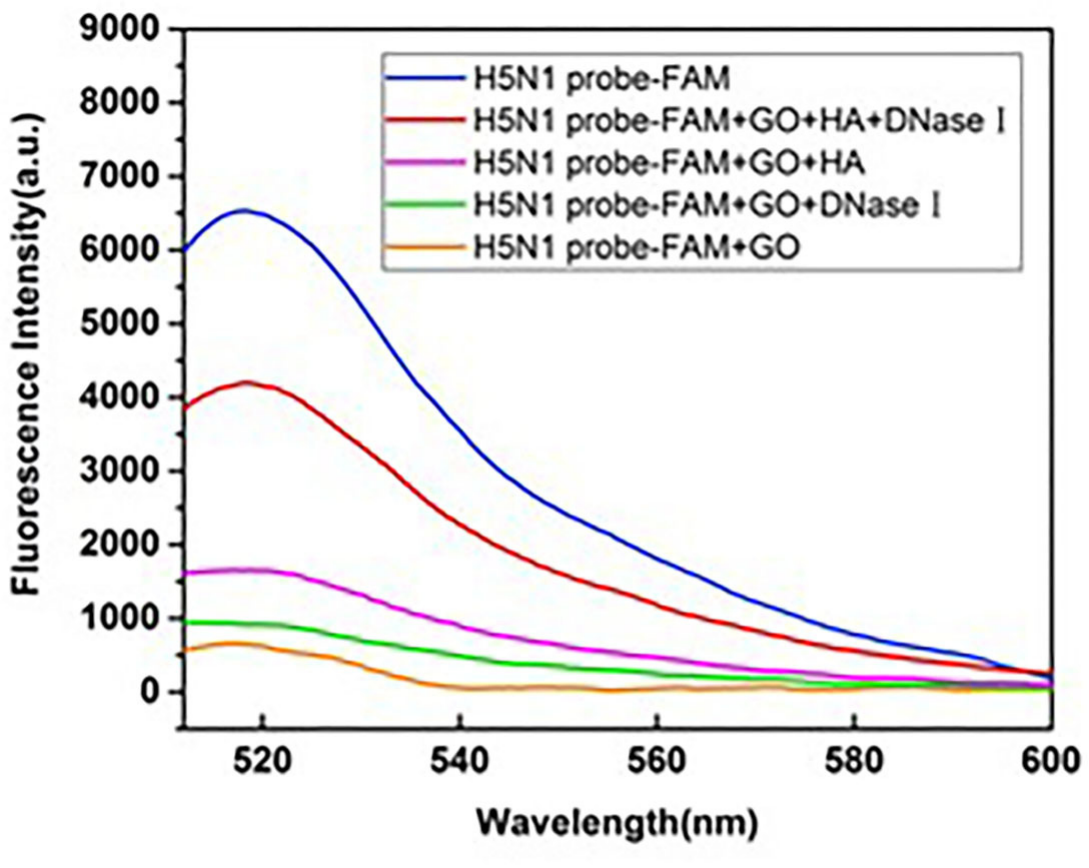
Feasibility of GO quenching and DNase I cleavage in the reaction system.

Taking the aptamer targeting HA of H5N1 as an example, the fluorescence spectrum was monitored to examine the amplification effect of DNase I. As shown in Figure 5, the single aptamer probe labeled with FAM was used as a positive control and a mixture of HA-H5N1 probe and GO as the negative control. Upon addition of DNase I to the negative control, the fluorescent signal displayed no obvious recovery, supporting a protective effect of GO against digestion. The negligible increase in fluorescence intensity was attributed to the background fluorescence of DNase I itself. In the presence of HA, the fluorescence signal was recovered to a certain extent. However, when DNase I was introduced in the above system, the fluorescence signal increased nearly doubled in intensity, indicating an amplification effect on aptamer signals.

### Optimization of the DNase I Amount and Incubation Time

**Figure 6:**
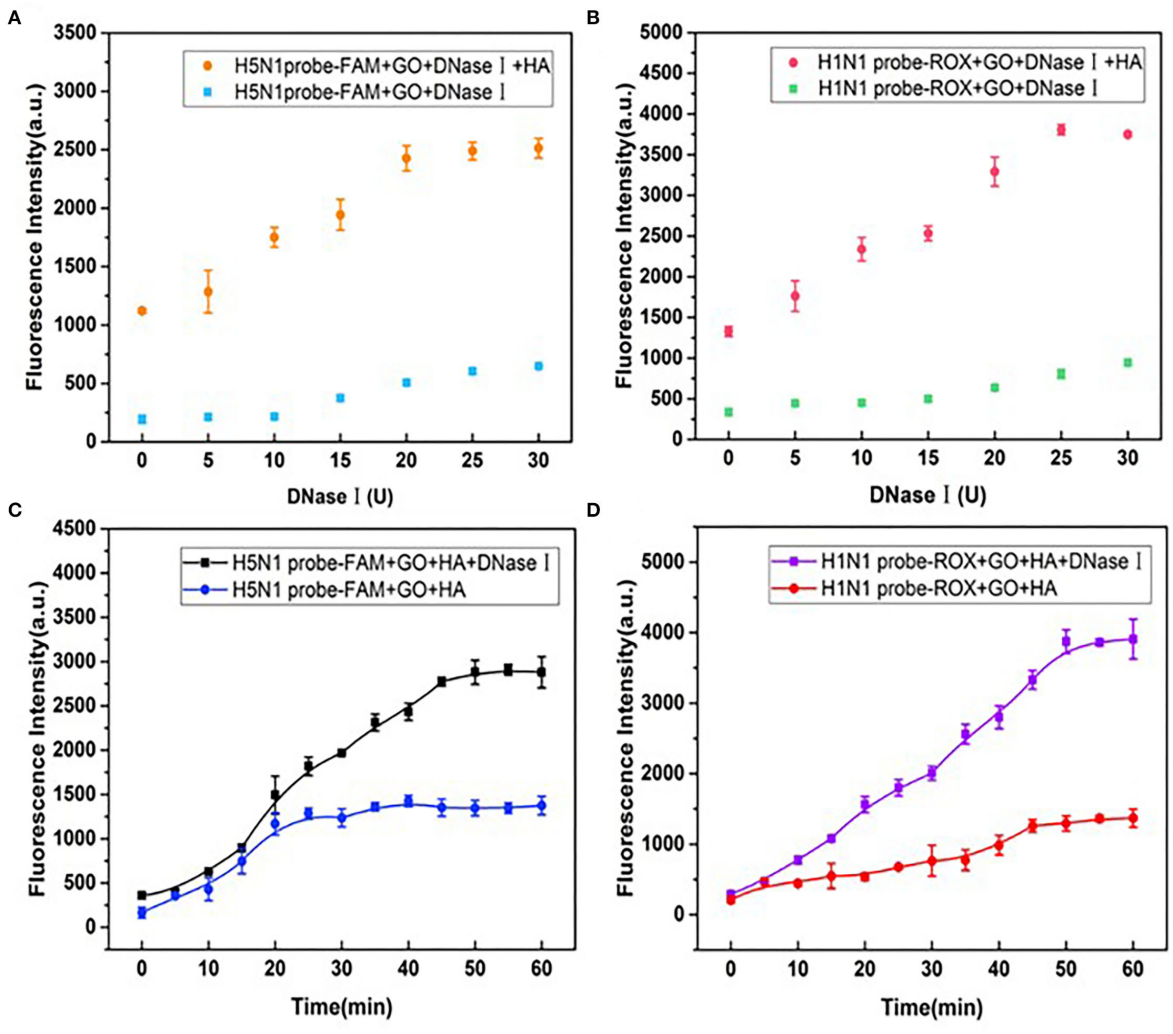
Effects of different doses of DNase I on fluorescence recovery for **(A)** H5N1-FAM and **(B)** H1N1-ROX probes in the detection system. Effects of incubation times of DNase I on fluorescence recovery for **(C)** H5N1-FAM and **(D)** H1N1-ROX probes.

The DNase I level is one of the critical parameters in high-sensitivity detection. Notably, the introduction of higher concentrations of DNase I increased background fluorescence. Fluorescence recovery reached maximal levels at DNase concentrations of 20 and 25 U for probes of H1N1 and H5N1, respectively (Figures 6A,B), and a dose of 25 U was thus used for subsequent experiments. In addition, the optimal incubation time of DNase I was determined. As shown in Figures 6C,D, in the presence of HA but absence of DNase I, fluorescence recovery for FAM and ROX plateaued at 30 and 45 min, respectively. In the presence of DNase I, fluorescence recovery was stable at 45 and 50 min, respectively. Accordingly, an optimal DNase I incubation time of 50 min was used for subsequent experiments.

### DNase I-Aided Signal Amplification for Dual Detection of HA Proteins From H5N1 and H1N1

**Figure 7:**
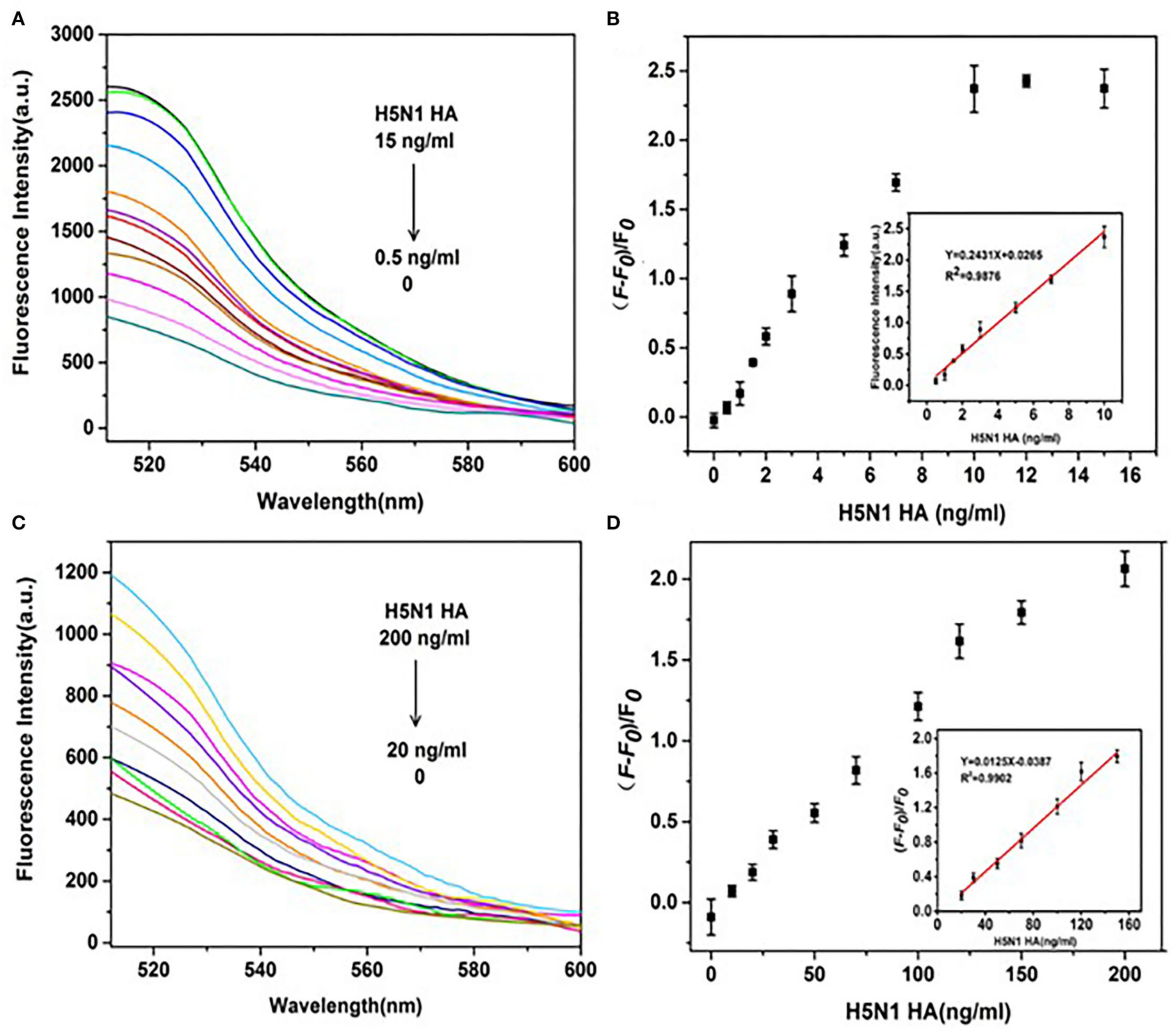
Effects of DNase I on the sensitivity of detection of H5N1 HA. **(A)** Fluorescence spectra of a series of concentrations of H5N1 HA (0, 0.5, 1, 1.5, 2, 3, 5, 7, 10, 12, and 15 ng/ml) in the presence of DNase I. **(B)** (F – F0)/F0(fluorescence change) vs. concentration of H5N1 HA in the presence of DNase I. **(C)** Fluorescence intensities of a series of concentrations of H5N1 HA (0, 10, 20, 30, 50, 70, 100, 120, 150, and 200 ng/ml) in the absence of DNase I. **(D)** (F – F0)/F0 (fluorescence change) vs. concentration of H5N1 HA in the absence of DNase I. The insets show a linear relationship between (F – F0)/F0 and H5N1 HA concentration in the presence of DNase I **(B)** and the absence of DNase I **(D)**.

To further improve the sensitivity of HA detection, the amplification effects of DNase I on fluorescence signals of HA from H1N1 and H5N1 under the established optimal conditions were investigated. First, fluorescence emission spectra of the aptamer probe upon addition of different concentrations of H5N1 HA were recorded. The fluorescence intensity without HA (F0) represented the background fluorescence due to GO quenching. Fluorescence intensity at the time of introduction of HA (F) in the reaction system was further monitored. The change in fluorescence was determined as (F – F0)/F0, representing the recovery degree of the fluorescence signal. In the presence of DNase I, within a range of 0.5–15 ng/ml H5N1 HA, the fluorescence intensity of FAM gradually increased in a dose-dependent manner (Figure 7A). The results shown in the inset of Figure 7B support a good linear relationship in the range of 0.5–10 ng/ml between fluorescence changes and the concentration of H5N1 HA. The linear equation was *y* = 0.2431*x* + 0.0265 (*R*^2^ = 0.988), with limit of detection (LOD) as low as 0.73 ng/ml (3 *s*/*n, n* = 11). In the control system without DNase I (Figures 7C,D), a linear relationship was observed in the range of 20–150 ng/ml. The linear equation was *y* = 0.0125*x* – 0.0387 (*R*^2^ = 0.990), with LOD as low as 20.17 ng/ml (3 *s*/*n, n* = 11). Comparative analysis showed that DNase I improved sensitivity up to 27 times relative to the DNase I-free system.

**Figure 8:**
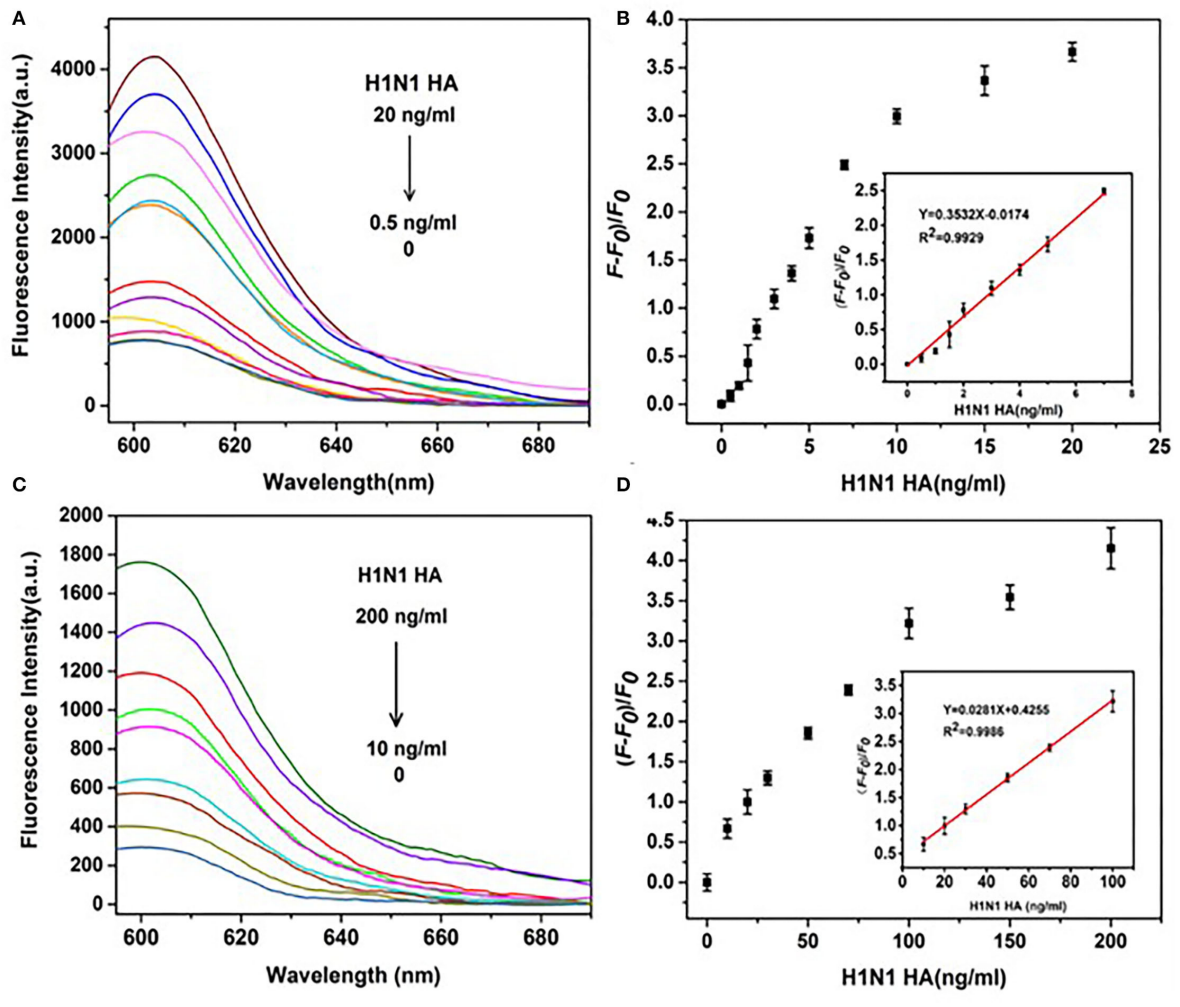
Effects of DNase I on the sensitivity of detection of H1N1 HA. **(A)** Fluorescence spectra of a series of concentrations of H5N1 HA (0, 0.5, 1, 1.5, 2, 3, 4, 5, 7, 10, 15, and 20 ng/ml) in the presence of DNase I. **(B)** (F – F0)/F0 (fluorescence change) vs. concentration of H1N1 HA in the presence of DNase I. **(C)** Fluorescence intensities of a series of concentrations of H1N1 HA (0, 10, 20, 30, 50, 70, 100, 120, 150, and 200 ng/ml) in the absence of DNase I. **(D)** (F – F0)/F0 (fluorescence change) vs. concentration of H1N1 HA in the absence of DNase I. The insets depict a linear relationship between (F – F0)/F0 and the concentration of HA H1N1 in the presence of DNase I **(B)** and the absence of DNase I **(D)**.

Comparable results were obtained for H1N1 HA using the same detection system. The fluorescence intensity gradually increased in a dose-dependent manner from 0.5 to 20 ng/ml and showed a linear relationship in the range of 0.5–7 ng/ml (Figures 8A,B) in the presence of DNase I. The linear equation was *y* = 0.3532*x* – 0.0174 (*R*^2^ = 0.993), with LOD as low as 0.43 ng/ml (3 *s*/*n, n* = 11). In the absence of DNase I, a linear relationship was observed in the range of 10–100 ng/ml (Figures 8C,D). The linear equation was *y* = 0.0281*x* + 0.4255(*R*^2^ = 0.999), with LOD of 8.22 ng/ml (3 *s*/*n, n* = 11). Amplification by DNase I significantly enhanced the sensitivity of detection of H1N1 HA by 19 folds. Overall, the detection system incorporating DNase I was able to simultaneously detect HA proteins of both H1N1 and H5N1 with significantly higher sensitivity.

### Specificity of the Aptamer Sensor for H5N1 and H1N1

**Figure 9:**
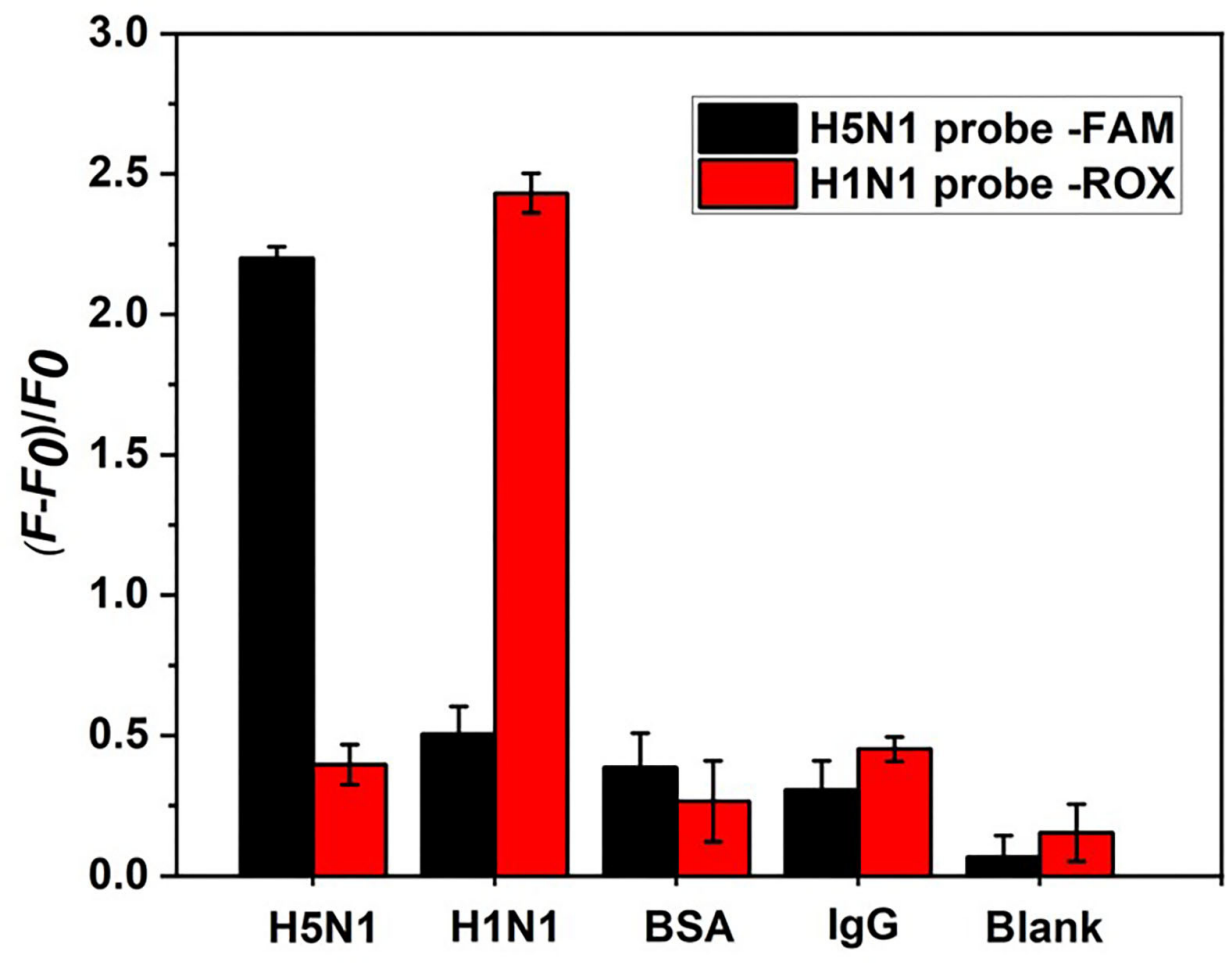
Specificity analysis of the aptamer sensor. HA proteins from H1N1 and H5N1 act as counterpoint controls for each other. Background control protein BSA and human IgG are at a concentration of 20 ng/ml.

Although the specificity of aptamers for single HA proteins from H1N1 or H5N1 influenza viruses has been confirmed (Cheng et al., 2008; Lao et al., 2014), aptamers used for dual detection of HA from both H5N1 and H1N1 require further analysis. Experiments were performed to compare the fluorescence signal changes produced by HA and other potential interfering proteins that were twice the concentration of HA. As shown in Figure 9, other protein types induced only a weak increase in the fluorescence signal of the H5N1 or H1N1 probe. These minor errors could be attributed to the non-specific replacement of protein and aptamer or its background fluorescence signal. The collective findings validated the specificity of our aptamer sensor for HA proteins from H5N1 and H1N1.

### Detection of HA in Serum Samples

The complex components of actual clinical samples may significantly interfere with the accuracy and sensitivity of detection. Five healthy human serum samples were filtered and diluted 50 times with binding buffer to simulate clinical samples (Table 1). As shown in Table 2, upon addition of different concentrations of HA from H1N1 and H5N1 to serum samples, recovery was 88.23–117.86% (*n* = 8) and the relative standard deviation for reproducibility could be controlled within 10%. The results support good selectivity and stability of the method for HA detection in clinical serum samples.

**Table 2:** Dual detection of the HA from H1N1 and H5N1 viruses in human serum samples.

**Sample**	**Added (ng/mL)**		**Found (ng/mL)**		**Recovery (%)**		**Reproducibility (%)**	
**Numbers**	**H1N1**	**H5N1**	**H1N1**	**H5N1**	**H1N1**	**H5N1**	**H1N1**	**H5N1**
	**HA**	**HA**	**HA**	**HA**	**HA**	**HA**	**HA**	**HA**
1	5	–	5.27 ± 0.34	–	105.49	–	6.9	–
2	–	5	–	4.92 ± 0.46	–	98.4	–	9.29
3	5	5	5.20 ± 0.15	5.89 ± 0.36	104.05	117.8	3	7.22
4	2	2	2.18 ± 0.15	1.87 ± 0.19	109.05	93.7	7.64	9.85
5	10	10	9.12 ± 0.46	8.82 ± 0.73	91.23	88.2	4.61	7.3

We additionally calculated the cost of detection of H1N1 and H5N1 using the aptasensor. The cost was US$1 per sample (experiments were repeated three times). It may be more cost-effective to purchase reagents in large quantities. The time of detection using our system was established as 1 h (including 5 min of GO and HA incubation and 50 min of DNase I incubation).

## Discussion

Influenza A virus infections are associated with varying morbidity and mortality rates. One of the currently circulating IAVs in humans is subtype A (H1N1), which is far less deadly. HP avian H5N1 influenza viruses have continued to spread throughout Asia and into Europe and Africa. These viruses cause high mortality rates and raise mounting international concerns of a looming pandemic. Highly sensitive systems that can simultaneously discriminate mild and HP subtypes of IAV may provide practical diagnostic guidance and help to reduce the pressure on the medical system.

Homogeneous assays possess several advantages, such as biochemical universality, inexpensive experimental conditions, operational convenience, robustness, and exceptional natural selectivity. FRET-based homogeneous assays are widely used to detect targets of biomolecules. In this study, an aptamer sensor capable of detecting HA from both H5N1 and H1N1 was established based on FRET. The FAM-labeled aptamer of HA from H5N1 and ROX-labeled aptamer of H1N1 HA were used as the energy donors. GO is a highly efficient energy acceptor due to its wide absorption spectrum (~300–700 nm). To avoid non-specific adsorption, PEG800 was selected as the blocking agent. Under optimum conditions, the detection range for HA from the H5N1 subtype was 20–150 ng/ml and LOD was as low as 20.17 ng/ml. For H1N1 HA, the detection range was 10–100 ng/ml and LOD was 8.22 ng/ml. To further improve the sensitivity of detection, DNase I was added to amplify the fluorescence signal based on its cleavage of free aptamer and consequent release of target HA for recycling. With the aid of DNase I, the detection limits of H5N1 HA and H1N1 HA were 0.733 and 0.427 ng/ml, estimated as 27 and 19 times improvement in sensitivity, respectively. DNase I was thus successfully utilized to achieve signal amplification. The recovery rate in human serum samples ranged from 88.23 to 117.86%, indicating that the newly developed aptasensor provides stable and accurate measurements in a complex detection environment.

Our study has a number of limitations that should be taken into consideration (Table 3). First of all, the organic fluorescent dyes FAM and ROX for labeling aptamers used in dual detection of HA from H5N1 and H1N1 have potential defects in light stability and photobleaching resistance. Recently, our group synthesized small (sub-20 nm) sandwich-structured upconversion nanoparticles with high energy transfer efficiency that achieved a lower LOD of H5N1 HA (60.9 pg/ml) (Zhao et al., 2021). Sensitivity and specificity could be further improved by integrating DNase I with upconversion nanoparticles to reduce the background of autofluorescence and enhance light stability labels. Afterward, while our system facilitated detection of both H1N1 and H5N1, analysis of the HP strain H7N9 should additionally be considered. Further studies are essential for the optimization of the system to simultaneously discriminate H1, H5, and H7 viruses. Finally, due to limitations in experimental conditions, clinical samples were not used for the examination of specificity and sensitivity.

**Table 3:** Comparison of biosensor detection of influenza virus.

**Biosensor**	**Method**	**Influenza type (subtypes)**	**Target**	**Detection limit**	**Time to result**	**Cost**	**Reference**
Biosensor (Nucleic acid direct detection)	Peptide nucleic acid biosensor	A	Gene M1	2.3 ng	11 min		Kumar et al., 2020
	Duo-genosensor with DNA probes	A(H5N1)	Gene HA NA	100 nM	>120 min		Grabowska et al., 2013
	Fluorescent aptasensor based on nanoparticles metal-enhanced fluorescence	A(H5N1)	Gene HA	2 ng/mL	–	–	Pang et al., 2015
Biosensor (protein detection)	Upconversion Fluorescence Resonance Energy Transfer Aptasensors	A(H5N1)	Protein HA	60.9 pg/mL	80 min	$15	Zhao et al., 2021
	Electrochemical impedance based diamond biosensor	A	Protein M1	1 fg/ml	5 min	–	Nidzworski et al., 2017
	An impedance aptasensor with gold nanoparticles	A(H5N1)	Protein HA	0.25 HAU	60 min	$3	Karash et al., 2016
	An Impedance Aptasensor with Microfluidic Chips	A(H5N1)	Protein HA	0.128 HAU	–	$100	Lum et al., 2015
	SPR aptasensor	A(H5N1)	Protein HA	0.128 HAU	95 min	–	Bai et al., 2012
	Hydrogel based a quartz crystal microbalance aptasensor	A(H5N1)	Protein HA	0.0128 HAU	–	$64	Wang and Li, 2013

## Conclusion

A signal-amplified aptamer sensor capable of dual detecting H5N1 HA and H1N1 HA was successfully constructed. The detection limits of H5N1 HA and H1N1 HA using this fluorometric method were 0.733 and 0.427 ng/ml, respectively. The sensitivity of our system was about 20-fold greater than that of traditional fluorometric methods without amplification. Furthermore, we observed no cross-reaction between the two influenza virus subtypes. The method showed good reproducibility and stability.

While we added HA protein into the serum to mimic clinical samples in this study, good reproducibility and stability of our aptasensor should facilitate the typing and early detection of influenza virus in real clinical samples in the future. The symptoms of infection of H5N1 and H1N1 are identical. Although H5N1 is HP, the incidence of transmission to humans is relatively low. Notably, H1N1 and H5N1 have high genetic compatibility and reassortment ability, posing a high risk of generation of pandemic strains. Our dual detection system for H1N1 and H5N1 may be effectively used for systematic surveillance of both influenza viruses to ensure early warning and preparedness for potential future pandemics.

## Data Availability Statement

The raw data supporting the conclusions of this article will be made available by the authors, without undue reservation.

## Ethics Statement

The studies involving human participants were reviewed and approved by the Ethics Review Committee of Tianjin University. The Ethics Committee waived the requirement of written informed consent for participation.

## Author Contributions

QZ, MH, and FL: performed the experiments. ZW: designed the study and wrote the manuscript. YD: processed the data. ZW and TW: contributed financial assistance. All the authors contributed to the article and approved the submitted version.

## Funding

This study was supported by the National Key Research and Development Program of China (grant no. 2017YFA0205102) and the Seed Foundation of Tianjin University (grant no. 2020XY-0078).

## Conflict of Interest

The authors declare that the research was conducted in the absence of any commercial or financial relationships that could be construed as a potential conflict of interest.

## Publisher's Note

All claims expressed in this article are solely those of the authors and do not necessarily represent those of their affiliated organizations, or those of the publisher, the editors and the reviewers. Any product that may be evaluated in this article, or claim that may be made by its manufacturer, is not guaranteed or endorsed by the publisher.
